# Nephrotoxicity of targeted therapy used to treat lung cancer

**DOI:** 10.3389/fimmu.2024.1369118

**Published:** 2024-07-04

**Authors:** Qiuling Li, Jieshan Lin, Guojun Hao, Aihua Xie, Shuangxin Liu, Bin Tang

**Affiliations:** ^1^ Department of Nephrology, Blood Purification Center, Zhongshan People’s Hospital, Zhongshan, China; ^2^ Department of Nephrology, Guangdong Provincial People’s Hospital (Guangdong Academy of Medical Sciences), Southern Medical University, Guangzhou, China

**Keywords:** lung cancer, targeted therapy, EGFR TKIs, ALK TKIs, nephrotoxicity

## Abstract

Lung cancer is the leading cause of cancer-related death worldwide, especially non-small cell lung cancer. Early diagnosis and better treatment choices have already provided a more promising prognosis for cancer patients. In targeted therapy, antagonists target specific genes supporting cancer growth, proliferation and metastasis. With the incorporation of targeted therapies in routine cancer therapy, it is imperative that the array of toxicities associated with these agents must be well-recognized and managed, especially since these toxicities are distinct from those seen with conventional cytotoxic agents. Drug-related nephrotoxicity has attracted attention when initiating cancer therapy. Our review aims to summarize the adverse renal effects caused by targeted therapy during lung cancer treatment, mainly focusing on EGFR and ALK tyrosine kinase inhibitors. Also, we discuss the possible mechanism of the side effect and provide managements to help improve the renal function in clinical practice.

## 1 Introduction

Lung cancer remains the leading cause of cancer-related death worldwide, however, many patients diagnosed with advanced disease at their initial presentation with a high mortality rate. Based on microscopic evidence and histological features, lung cancer can be divided into three main subtypes: non-small cell lung cancer (NSCLC), small cell lung cancer (SCLC), and lung carcinoid tumors, of which non-small cell carcinoma accounts for up to 85% (1). Adenocarcinoma, squamous cell carcinoma and large cell carcinoma are the three most common forms of NSCLC (2). Lung cancer-related deaths accounted for 18.4% of total cancer deaths in 2018. More than 2000,000 new cases of lung cancer are expected to emerge, and up to 1761007 patients reach the death endpoint (3). The factors affecting the occurrence and progression of lung cancer mainly include smoking, air pollution, occupational exposure and genetic factors. Surgery, chemotherapy and radiation therapy are the traditional treatment choices for lung cancer. However, due to lack of specificity, chemotherapy and radiation therapy have also caused adverse effects such as bone marrow suppression, gastrointestinal reactions and local inflammation for cancer patients (4, 5).

Recent years, with the development of molecular biology technology, targeted therapies have provided us with more promising options for treating these diseases (6). Recent research has confirmed that folate-bovine serum albumin-coated ethoniosomes of pterostilbene allowed specific targeting of cancer tissues overexpressing folate receptors rather than healthy tissues. Since propolis is rich in a variety of different polyphenolic compounds, it can enhance antioxidant activity and regulate signaling pathways in cancer cells, and the propolis extract is loaded into albumin-folic acid to minimize the strong smell and taste of propolis and control its delivery, which in turn targets cancer cells (7, 8). For stage IVB cancer that has metastasized throughout the body, the cancer cells need to be tested for the presence of specific genetic mutations before any treatment. Correct screening of gene mutations in tumor patients is the premise of an individualized treatment plan. Compared with traditional treatment, targeted therapy has significantly improved the objective response rate (ORR) and progression free survival (PFS) of mutation-positive NSCLC, therefore the progress in molecular pathogenesis research of lung cancer is of great significance to targeted therapy with specific molecular changes (9, 10). Common gene mutations in lung cancer include the epidermal growth factor receptor (EGFR), vascular endothelial growth factor (VEGF), anaplastic lymphoma kinase (ALK), c-ros oncogene 1 (ROS1), Kirsten rat sarcoma viral oncogene homolog (KRAS), serine/threonine-protein kinase b-raf (BRAF), proto-oncogene tyrosine-protein kinase receptor Ret (RET), mesenchymal epithelial transition factor (MET). The EGFR mutation and ALK mutation are the most frequent oncogenic driver of NSCLC (11). We summarized these two molecular targeted therapies and common adverse events in **Table 1**.

**Table 1:** Application, molecule target and common adverse events of EGFR and ALK TKIs.

Drug Class	Drug name	Mechanism and applications	Common molecular target	Common adverse events
EGFR TKIs	Erlotinib	1st-generation; Reversible binding to WT and mutant EGFR; Can be used in combination with VEGF antibodies, such as bevacizumab	EGFR L858R, Del 19	Rash, diarrhea, hepatotoxicity and interstitial lung disease
	Gefitinib	1st-generation; Reversible binding to WT and mutant EGFR	EGFR L858R, Del 19	Rash, diarrhea, hepatotoxicity and interstitial lung disease
	Afatinib	2nd-generation; Irreversible covalent binding to EGFR, HER2 and HER4 to inhibit all HER family signaling; More preferred in squamous NSCLC or brain metastasis;	EGFR L858R, Del 19; G719X, S768I, and L861Q; Wild type-HER2, HER2 amplification, HER4	Rash, diarrhea, hepatotoxicity and interstitial lung disease
	Osimertinib	3rd-generation; Irreversible covalent binding to mutant EGFR; Specificity for EGFR T790M mutant; More preferred in brain metastasis;	EGFR L858R, Del 19; EGFR T790M	Less frequent adverse events
ALK TKIs	Crizotinib	1st-generation;	EML4-ALK; MET, ROS1	Visual impairment, gastrointestinal disorders
	Ceritinib	2nd-generation; Higher potent;	EML4-ALK; ALK C1156Y, I1171T, F1174C, L1198F, D1203N, E1210K, G1269A	Gastrointestinal and hepatic disorder, more frequent AEs
	Alectinib	2nd-generation; First choice for first-line treatment of ALK-positive NSCLC; More preferred in brain metastasis;	EML4-ALK; ALK C1156Y, I1171N/S/T, F1174C, L1196M, D1203N, E1210K, G1269A	Gastrointestinal and hepatobiliary disorders
	Brigatinib	2nd-generation;	EML4-ALK	Gastrointestinal disorder, fatigue, headache
	Lorlatinib	3rd-generation;	EML4-ALK; ROS1 G2032R	Neurocognitive impairments

EGFR, epidermal growth factor receptor; ALK, anaplastic lymphoma kinase; TKI, tyrosine kinase inhibitor; WT, wild type; NSCLC, non-small cell lung cancer; VEGF, vascular endothelial growth factor, HER, human epidermal growth factor receptor; EMLK4, echinoderm microtubule-associated protein-like 4; ROS1, c-ros oncogene 1.

The kidneys perform a vital role in the proper functioning of the body and in maintaining homeostasis. The kidneys excreted endogenous metabolic products as well as eliminated drugs and toxins. Nephrotoxicity is a common complication during many medical procedures, including those used for cancer treatment. Both chemotherapy and immunotherapy may cause deterioration of kidney function, which in turn leads to increased mortality in cancer patients. Antineoplastic drugs may damage the glomeruli, renal tubules or any other part of the nephron, leading to deterioration of renal function and the appearance of various clinical symptoms such as acute kidney injury, electrolyte disturbances, nephrotic syndrome and glomerulonephritis (12, 13). Previous review has usually focused on nephrotoxicity caused by a particular series of drugs or has not explored the specific mechanism and clinical management. In this review, in addition to the extensive clinical studies, we also included case reports and case series, and summarized different types of small molecule targeted drugs to provide effective clinical management by exploring the possible mechanisms of nephrotoxicity. In **Table 2**, we have summarized the details of approved EGFR and ALK-targeted therapies along with dosing in CKD patients. In this review, we aim to summarize the current literature on the nephrotoxicities of small molecule inhibitors, especially EGFR and ALK tyrosine kinase inhibitors, and provide possible managements of the renal adverse effect.

**Table 2:** Approved EGFR and ALK TKIs along with dosing in CKD.

Drug class	Drug name	Approved Dosage (mg)	Clearance	Renal excretion	Dose adjustment for mild to moderate CKD	Dialysis dose adjustment
EGFR TKIs	Erlotinib	150 P.O. QD	Via the feces	<9%		No (caution needed)
	Gefitinib	250 P.O. QD		<4%		
	Afatinib	40 P.O. QD		<5%	No	
	Osimertinib	80 P.O. QD		14%		
ALK TKIs	Crizotinib	250 P.O.BID	Via the feces	No	No	No (caution needed)
	Ceritinib	750 P.O. QD				
	Alectinib	600 P.O.BID				
	Brigatinib	90 P.O. QD				
	Lorlatinib	100 P.O. QD				

EGFR, epidermal growth factor receptor; ALK, anaplastic lymphoma kinase; TKI, tyrosine kinase inhibitor; AKI, acute kidney injury; P.O., orally; QD, once per day; BID, twice a day; NSCLC, non-small cell lung cancer.

## 2 EGFR tyrosine kinase inhibitors

The EGFR belongs to the human epidermal growth factor receptor (HER) family. The physiological function of EGFR involves the development of epithelial tissue and maintenance of homeostasis. Dysregulation of these tyrosine kinases and their downstream signaling pathways is associated with cancer cell proliferation, angiogenesis, and metastasis (14). After ligand binding, EGFR tyrosine kinase activates the receptor by homologousing or heterodimering it and auto-phosphorylating the tyrosine-rich cytoplasmic region, which initiates two main downstream intermediate pathways (15). EGFR mutations account for nearly 10% to 60% in patients of NSCLC (16). EGFR mutations occur predominantly in exons 18-21 encoding the intracellular domain of tyrosine kinase. The in-frame deletion of exon 19 and the L858R missense mutation of exon 21 are the most common activating mutations in EGFR, accounting for more than 90% of the totals (17). Uncommon EGFR mutation, including G719X at exon 18, S768I at exon 20, and L861Q at exon 21 have reported in NSCLC patients (18). EGFR inhibitors, mainly including monoclonal antibodies and tyrosine kinase inhibitors, are one of the widely used targeted therapies in oncology (19). These drugs work by specifically targeting and inhibiting the EGFR, a type of protein found on the surface of some cancer cells that can promote cell growth, multiplication and angiogenesis. The development of these drugs has already significantly improved the prognosis of patients. EGFR tyrosine kinase inhibitors (TKIs), mainly including afatinib, erlotinib and gefitinib, are suitable for treating NSCLC harboring EGFR mutations. Erlotinib and gefitinib are widely used as the first-generation of anti-EGFR drugs, however, as the resistance to the treatment, the second-generation anti-EGFR drug, afatinib and the third-generation osimertinib, which targets the EGFR mutation T790M (20, 21). Erlotinib and gefitinib are reversibly inhibit EGFR, whereas afatinib is irreversible covalent binding to EGFR, HER2, and HER4 to inhibit all HER family signaling with a broader activity to overcome EGFR TKI-resistant mutations (22). Rash, diarrhea, hepatotoxicity, and less commonly, but important, interstitial lung disease are common adverse events (AEs) in clinical practice of first and second-generation of EGFR TKIs. About 6.1% of patients suffered treatment withdrawal due to the unpleasant adverse events, especially skin disorders, interstitial lung disease and hepatoxicity, but less frequent reported in osimertinib (23, 24).

In the kidney, EGFR was mainly expressed in renal tubules, especially in distal and collecting ducts, and to a lesser extent in glomerular capillary walls, proximal tubules and mesangial cells (25). EGF is essential in maintaining the integrity of renal tubules. EGFR activation results in the growth and regeneration of renal tubular epithelial cells after acute tubular necrosis (26). Renal side effects caused by EGFR tyrosine kinase inhibitors can differ among patients but commonly include electrolyte disturbances, such as hypomagnesemia, hypokalemia and hypophosphatemia. Also, proteinuria, hypertension, and acute kidney injury may occur, though uncommon, still reports showed indirect renal toxicity caused by diarrhea-induced dehydration may happen and it is pretty essential to recognize timely and take managements (27–29). **Table 3** and **Table 4** respectively summarizes the renal toxic events and possible mechanism of EGFR inhibitors in lung cancer. And the clinical managements have been listed in detail in **Table 4**.

**Table 3:** Summary of renal toxic events with EGFR and ALK targeted agents.

Drug class	Drug name	Renal adverse events
EGFR TKIs	Erlotinib	AKI, hypomagnesemia, hypophosphatemia
	Gefitinib	AKI, nephrotic syndrome, membranous nephropathy, hypokalemia, fluid retention
	Afatinib	AKI, hypokalemia, hyponatremia
	Osimertinib	AKI, rhabdomyolysis
ALK TKIs	Crizotinib	Elevated serum creatinine, renal cyst formation
	Ceritinib	Hypophosphatemia, hypomagnesaemia, hyponatremia
	Alectinib	AKI, hypophosphatemia
	Brigatinib	Tumor lysis syndrome
	Lorlatinib	Diffuse edema, nephrotic syndrome

EGFR, epidermal growth factor receptor; ALK, anaplastic lymphoma kinase; TKI, tyrosine kinase inhibitor; AKI, acute kidney injury.

**Table 4:** Clinical management and possible mechanism to renal adverse events of EGFR and ALK targeted agents.

Drug class	Adverse events	Possible mechanism	Clinical management
EGFR TKIs	Electrolyte disorders	Hypomagnesemia: insufficient activation of TRPM-6 type channel Hypophosphatemia: dysfunction sodium phosphate co-transporters in the proximal tubule	Holding the dose; Regularly monitor electrolyte levels and kidney function, especially serum magnesium and phosphorus.
	Elevated serum creatinine	Acute tubular necrosis; rhabdomyolysis	Regularly monitor serum creatinine and creatine kinase.
	Proteinuria	Unknown, possible an allergic or immune reaction	Holding original dose or reduce the dose; Closely monitored with regular urine test; Renal biopsy
ALK TKIs	Renal cyst formation	Activation of the HGF/MET signaling axis; Imbalance of testosterone hormone levels;	Usually self-limiting and holding the dose; Closely monitored with regular imaging
	Peripheral edema	Inhibition of c-MET pathway; Late-onset cumulative effect	Low-grade edema, compression stockings, leg elevation, and lifestyle changes; Diuretics only in severe edema
	Electrolyte disorders	Hypophosphatemia: Inhibition of the insulin (IGF-1) receptor; Hyponatremia: inadequate secretion of antidiuretic hormone	Holding the dose; Regularly monitor electrolyte levels and kidney function, especially serum magnesium and phosphorus
	Proteinuria	Likely podocytopathies	Holding original dose or reduce the dose; Closely monitored with regular urine test; Renal biopsy
	Elevated serum creatinine	Pseudo-kidney injury due to creatinine transporter inhibition; Inhibition of c-MET pathway; acute tubular necrosis	Check cystatin-C based glomerular filtration to rule out pseudo-AKI; otherwise, drug discontinuation and closely check cystatin-C based glomerular filtration

EGFR, epidermal growth factor receptor; ALK, anaplastic lymphoma kinase; TKI, tyrosine kinase inhibitor; AKI, acute kidney injury; eGFR, estimated glomerular filtration rate; MET, mesenchymal epithelial transition factor.

### 2.1 Erlotinib

Erlotinib is the first-line treatment for patients with locally advanced or metastatic EGFR mutations with NSCLC, primarily as maintenance therapy for these patients (30). Erlotinib can be used in combination with VEGF antibodies, such as bevacizumab (31). The usual dose of erlotinib is 150 mg orally per day, and the drug binds to plasma proteins up to 95%. Its metabolites are excreted mainly in feces, while the proportion of renal elimination is less than 9% (32). FDA Adverse Event Reporting System (FAERS) review revealed that 63 patients with acute kidney injury and 8 patients developed hypomagnesemia (33). Recently, Crosnier et al. (27) indicated that 139/303(45.9%) patients present with acute kidney injury among the patients with lung cancer (271/303) by reviewing the VigiBase^®^ the WHO Global Database, and diarrhea-related acute kidney injury was the leading cause.

**Figure 1:**
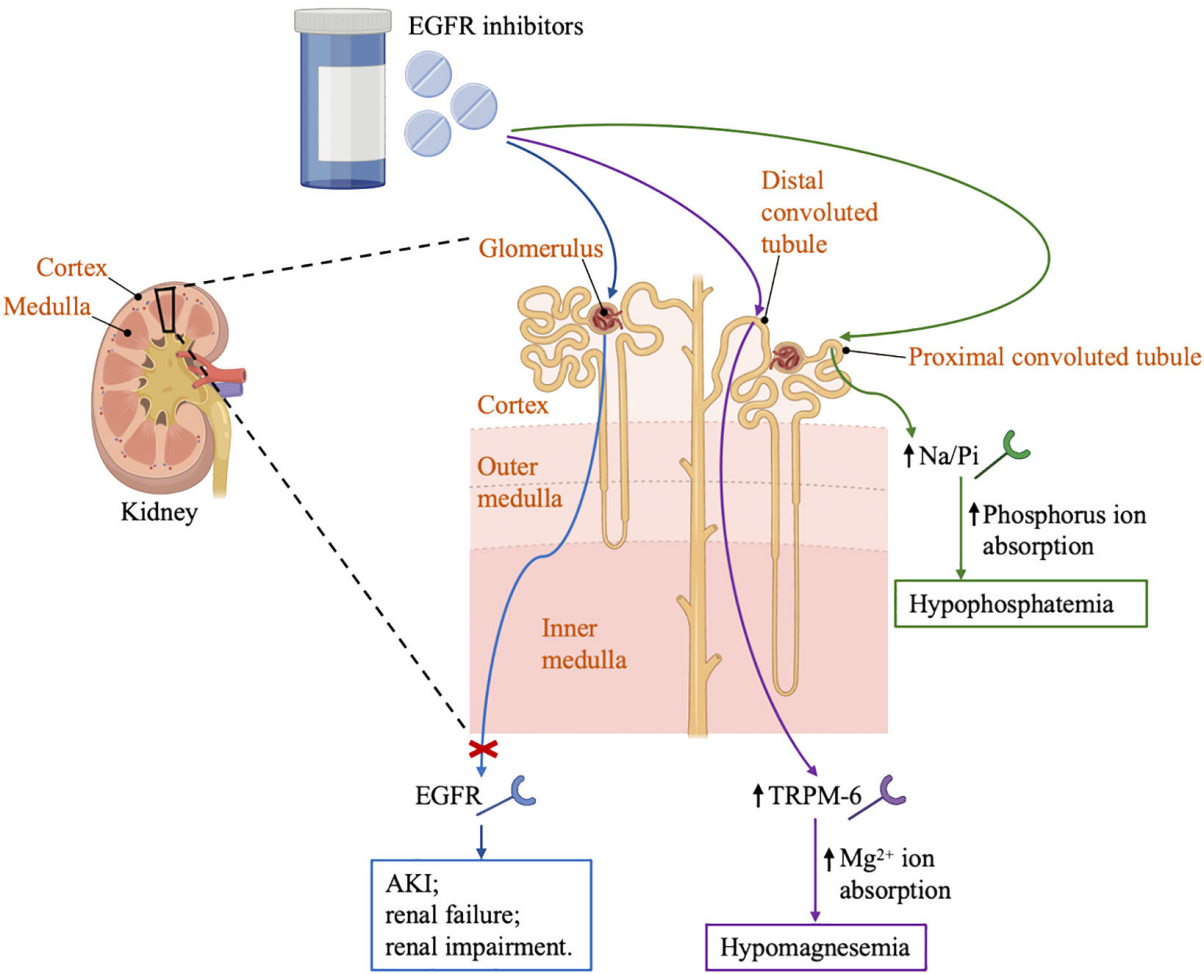
Renal effects of EGFR inhibitors. EGFR, the epidermal growth factor receptor; AKI, acute kidney injury; TRPM6, transient receptor potential cation channel; Na/Pi, sodium-phosphate co-transporter; GBM, glomerular basement membrane.

In a small clinical trial of 17 patients with solid tumors treated with erlotinib and sorafenib, the incidence of hypophosphatemia was 76%. In comparison, another clinical trial that enrolled 25 patients diagnosed with glioma showed a 30% incidence of hypophosphatemia (34, 35). Erlotinib can affect magnesium hemostasis, but its effect on systemic magnesium concentrations does not appear to be as good as that observed in antibody-based EGFR inhibitors, and the erlotinib-induced hypomagnesemia may be corrected by magnesia supplementation (13). There are several proteins in the distal convoluted tubules that have been implicated in the transport of magnesium, including magnesium-permeable transient receptor potential cation channel, subfamily M, member 6 (TRPM6) and transient receptor potential cation channel, subfamily M, member 7 (TRPM7) (36). EGF is a magnesiotropic hormone that regulates magnesium levels by regulating the TRPM6 type channel. Mutation of EGF gene resulted in impaired basolateral sorting of pro-EGF, thereby the renal EGFR is inadequately stimulated, inducing insufficient activation of TRPM6 type channel and thus hypomagnesemia occurred, as described in **Figure 1** (37).

Therefore, it is recommended that all patients have their magnesium levels checked before starting treatment and then every 2 to 4 weeks, especially those with heart disease history. The severity of hypomagnesemia is closely related to treatment. Generally, EGFR TKIs induced hypomagnesemia may be corrected by extra magnesium supplementation. Hypomagnesemia can be divided into 5 grades according to the magnesium level (Grade 1 is 0.73-0.50 mmol/L, Grade 2 is 0.50-0.40 mmol/L, Grade 3 is 0.40-0.30 mmol/L, Grade 4 is <0.30 mmol/L, Grade 5 is death) (38). Grade 1 hypomagnesemia requires close monitoring of magnesium levels without medication. In grade 2 hypomagnesemia, magnesium supplementation, usually given intravenously with a dose of 4 grams of magnesium sulfate weekly, is highly recommended to avoid fatal arrhythmias, especially for those ineffective and poorly tolerated to oral magnesium supplementation (32, 39). Patients with grade 3/4 hypomagnesemia are advised to take 6-10 grams of magnesium sulfate twice a day twice a week, and monitor serum magnesium every other day until a stable state is reached. Another strategy may be to consider 2-month discontinuation of magnesium sulfate therapy in patients requiring frequent magnesium sulfate infusions (39).

In addition, hypophosphatemia has been reported in patients treated with EGFR inhibitors. Possible mechanism related to hypophosphatemia might involve sodium phosphate co-transporters in the proximal tubule (39). In general, hypophosphatemia should not be considered as a reason to discontinue aggressive cancer treatment unless it is recurrent and life-threatening, especially given that the use of these drugs may significantly improve life expectancy in many cancer patients. Intravenous rehydration should be considered in any symptomatic or severely exhausted patient (phosphate <1.0 mg/dL). Oral replacement therapy can be resumed after the patient has resolved symptoms or serum phosphate > 1.5 mg/dL (40). Therefore, it is advisable to check electrolyte levels before initiation of therapy, and then routinely monitor to prevent the development of severe complications caused by electrolyte disorders.

Erlotinib-related acute and chronic kidney dysfunction, though uncommon, still occur in clinical practice. Even for the same type of drug, patients may respond differently due to the different chemical structure of the drug. Maruyama et al (41) described that a 57-year-old patient with advanced lung adenocarcinoma developed nephrotic syndrome after taking gefitinib, which improved after discontinuation and switched to erlotinib without any kidney damage, and possible explanation is related to drug hypersensitivity reactions. Besides, another case reported that patient had only mild proteinuria on erlotinib, but developed nephrotic range proteinuria on gefitinib (42). While all EGFR TKIs have the potential to cause direct damage to glomerular podocytes, these drugs may also produce podocyte damage through indirect pathways. Different pathways of injury may explain the severity of podocyte injury in these EGFR TKIs (42). However, more researches are needed to explore the precise mechanism. Therefore, it is highly recommended to check the renal function before erlotinib therapy, and then check routinely for early recognition and treatment to prevent the development of severe complications.

### 2.2 Gefitinib

Gefitinib is another TKI that blocks the activity of the EGFR tyrosine kinase. Gefitinib is used across all lines of therapy for patients with locally advanced or metastatic NSCLC with activating mutations of EGFR (20, 28). The usual dose of gefitinib is 250 mg orally per day, and the drug binds to plasma proteins up to 90%. Its metabolites are excreted mainly in feces, while the proportion of renal elimination is less than 4% (32). The most common adverse effect caused by gefitinib included diarrhea, vomiting and skin rash. Also, renal dysfunction of gefitinib were observed in clinical studies, with literature reviewed that 6.6% patients with gefitinib therapy developed fluid retention (43, 44). According to the FAERS, 7 patients after gefitinib initiation developed acute kidney injury, with hypokalemia and hyponatremia as the second and third adverse renal effects (33).

Pathological manifestations, including minimal change and mild IgA deposition, crescent formation, and tubular injury under microscopy have been reported in patients treated with EGFR inhibitors. A possible mechanism was proposed as an allergic or immune reaction as mild interstitial infiltration of lymphocytes was noted under the renal biopsy (44, 45). Kumasaka et al (44) reported a 76-year-old male presented with nephrotic syndrome after gefitinib therapy. Renal biopsy confirmed minimal change disease under electron microscopy, and her renal function discovered after discontinuation of gefitinib. Another case of acute renal failure due to thrombotic thrombocytopenic purpura/hemolytic uremic syndrome caused by gefitinib has been also reported. A 73-year-old male without potential precipitating factors for renal insufficiency has become uremic and acidosis after 16-day therapy of gefitinib. His renal function improved rapidly after gefitinib discontinuation (45). Kaneko et al (46) described the first case of gefitinib-related membranous nephropathy. Possible mechanisms include that gefitinib can act as a hapten attached to some native proteins, triggering an antibody response. Through long-term use, gefitinib may produce autoimmune reactions. In addition, gefitinib can locally bind to the increased phosphorylation of EGFR on podocytes, change its structure and induce antibodies, and thereby lead to subepithelial immune complexes (44, 46). During the first few weeks of gefitinib treatment, close monitoring of kidney function is required. When renal insufficiency is found, gefitinib should be discontinued immediately, and efforts should be made to find other secondary factors, and the next treatment should be determined until the cause is identified.

### 2.3 Afatinib

Afatinib, an orally administered irreversible EGFR TKI, is the first-line treatment for patients with EGFR mutation-positive advanced squamous NSCLC and patients harboring uncommon EGFR mutation, including G719X, S768I, and L861Q mutation (18, 47). Studies have shown that compared to first-generation EGFR TKIs, afatinib has a more favorable outcome, with significantly prolonged progression-free survival and time to treatment failure (47, 48). The recommended dose of afatinib is 40 mg orally per day. The drug binds mainly to plasma proteins and is excreted in feces, the proportion of which is excreted in the urine does not exceed 5% (32). Pharmacokinetic data indicate that in patients with mild or moderate renal impairment, the dosage of afatinib does not need to be adjusted, but 30 mg orally is prescribed to patients with severe renal impairment, even those patients under hemodialysis (49). In preliminary trials of afatinib, the incidence of hypokalemia was 34% (50). 26 patients under afatinib therapy developed acute kidney injury was noted in the FEARS report, which was followed by hypokalemia and hyponatremia (33). Koch et al (51) reported a case of hyponatremia in a 58-year-old female patient with EGFR-positive lung adenocarcinoma treated with afatinib. Serum electrolytes should be monitored closely in patients treated with EGFR TKIs and drugs should not be discontinued unless the symptoms are very severe.

### 2.4 Osimertinib

Osimertinib, a third-generation EGFR TKI, has been developed due to the acquired T790M mutation caused by resistance for advanced NSCLC EGFR mutant patients progressed after first-line EGFR TKI therapy (52). The T790M mutation is the most common mechanism of acquired resistance in NSCLC treatment, accounting for 50-60% of secondary resistance after first-line therapy (53). Usually, administration with osimertinib 20 mg once per day is chosen as the initiating dose, which is sufficient to inhibit EGFR T790M, while doses equivalent to 80 mg or more are expected to lead to profound inhibition of tumor growth (52). A clinical trial for patients with EGFR inhibitor-resistant NSCLC at doses of 20 to 240 mg once daily revealed that diarrhea is the most common toxicity, followed by rash, nausea, and decreased appetite (24). Research has found that adverse effect is more common in patients with impaired renal function, however, under close monitor, osimertinib can be safely administered to cancer patients undergoing regular hemodialysis (54). In another clinical trial with locally advanced or metastatic, MET-amplified, EGFR mutation-positive NSCLC, a case of acute renal failure was found in patients treated with osimertinib plus savolitinib (55). Recently, more cases related to renal toxicities were reported. Niitsu et al (56) reported a patient with NSCLC developed acute kidney injury associated with biopsy-proven mild IgA deposition, crescent formation, and tubular injury after initiation of osimertinib and his renal function recovered after reducing the dose of osimertinib (from 80 mg/day to 40 mg/day). Another case reported that a patient with advanced lung adenocarcinoma presented with myalgia, muscular weakness after 5-month treatment with osimertinib and bevacizumab. He was diagnosed with osimertinib-associated rhabdomyolysis and developed acute renal insufficiency, hyperuricemia, metabolic acidosis and electrolyte disorders. However, all symptoms recovered after discontinuation of osimertinib (57). Few cases of osimertinib-related kidney dysfunction were reported and more attention and follow-up are required.

## 3 ALK inhibitors

Besides EGFR tyrosine kinase, ALK has been another most frequently identified mutational driver of NSCLC. Due to chromosomal inversion, part of the ALK gene is fused with the echinoderm microtubule-associated protein-like 4 (EML4) gene in a small proportion of NSCLC patients, resulting in the activation and transformation of the EML4-ALK fusion protein compositionally, resulting in oncogene addiction. EML4-ALK fusion and other ALK rearrangements occur in 3-7% of patients with NSCLC. ALK inhibitors have already greatly improved prognosis in patients with advanced ALK-positive NSCLC (58, 59). To date, crizotinib and ceritinib are the ALK inhibitors most commonly used in clinical practice to treat advanced NSCLC (59). However, in clinical practice, most patients develop tolerance within 1 year of initiating crizotinib therapy. Compared to crizotinib, second-generation ALK TKIs, including alectinib, ceritinib, and brigatinib, have better sensitivity to ALK-positive NSCLC harboring a secondary mutation such as L1196M or G1269A (60).

With the widespread use of ALK inhibitors, the adverse events associated with these drugs have gradually attracted attention. A recently meta-analysis revealed that adverse events happened in most participants under ALK inhibitors therapy, among them serious adverse events occurred over 20%. Most of the adverse events of ALK TKIs are grade 1 to 2, which are generally tolerated by most patients. Diarrhea, vomiting, liver dysfunction, and vision disorder are common adverse events of ALK TKIs. Visual impairment and gastrointestinal symptoms, such as nausea and vomiting, were frequent adverse events of crizotinib. The most common renal adverse events include renal cysts, peripheral edema, elevated serum creatinine and proteinuria (61, 62). According to FAERS analysis, ALK inhibitors can cause acute or chronic renal insufficiency and may also cause electrolyte abnormalities such as hyponatremia and hypophosphatemia (33). **Table 3** and **Table 4** respectively summarizes the renal toxic events and possible mechanism of ALK inhibitors in lung cancer. Besides, the clinical managements have been listed in detail in **Table 4**.

### 3.1 Crizotinib

Crizotinib, the first-generation oral small molecule targeted therapy, inhibits multiple tyrosine kinase inhibitors, especially ALK, MET and ROS1. It has been shown to significantly improve outcomes compared with chemotherapy in both first-line and subsequent treatment in patients with ALK-positive advanced NSCLC. The recommended dose of crizotinib is 250 mg twice orally per day (63, 64).

According to the FDA, about 4% of patients develop complex renal cysts after initiation of crizotinib. Lin et al (65) revealed significant changes in renal cysts in 7 patients (22%) among 32 patients treated with crizotinib for ALK-positive advanced NSCLC, however, these may reverse after crizotinib discontinuation. An analysis of 255 patients treated with crizotinib for at least 6 months showed that 9% of patients acquired new cysts, and 2% of patients with pre-existing cysts developed new cysts and acquired enlargements in existing cysts (66). A retrospective study of 60 patients treated with crizotinib identified that female (*p*=0.008) and the presence of renal cysts on baseline scan (*p*=0.044) significantly related with renal cyst development or growth during initiation of crizotinib therapy (67). Also, Asians, especially Koreans tend to have higher odd ratios of developing de no renal cysts during crizotinib treatment (66).

Another adverse renal effect related to crizotinib is acute kidney injury. Camidge et al (68) have demonstrated that elevated serum creatinine with crizotinib occurs mainly in the first 12 weeks after initiation of crizotinib. There is little evidence of cumulative effects with long-term treatment, and renal function is largely restored after discontinuation within one week, suggesting that this may be primarily an effect on creatinine secretion rather than true nephrotoxicity, which is named pseudo acute kidney injury.

However, there were still case reports related to actual kidney injury. Gastaud et al (69) reported a case that a 49-year-old patient with previously normal renal function developed AKI after 3 weeks of crizotinib treatment, and renal function returned to normal on day 8 after discontinuation and renal biopsy showed diffuse acute tubular injury and tubular necrosis. Another case described by Izzedine et al (70) showed a patient diagnosed with NSCLC along with chronic kidney disease presented with progressive renal function worsening after 11-month crizotinib therapy, with kidney biopsy revealing features of acute tubular injury without interstitial cell infiltration and renal arteriolar myocyte vacuolization.

Electrolyte disorders have also occurred during clinical practice, including hypophosphatemia, hyponatremia, hypokalemia and hypocalcemia. Among them, hypophosphatemia has come to the first as a serious adverse event with up to 15% of all participants reporting during the treatment of crizotinib (71). Researches have revealed that hypophosphatemia is a sensitive prognostic factor related to the in-hospital day, severe complications or all-cause mortality. Still, the exact mechanism for these adverse events remains unclear, and there are no formal recommendations for guidance. Possible explanation for hypophosphatemia is associated with inhibiting the insulin-like growth factor-1 (IGF-1) located in the proximal tubules blocking the phosphate reabsorption, thus resulting in phosphaturia (40).

Renal cyst formation has been reported in patients treated with ALK inhibitors, especially those treated with the first-generation crizotinib. Yet, the potential causal relationship between crizotinib treatment and renal cyst formation has not been fully established. Hepatocyte growth factor receptor (HGF) is the only known ligand for MET and plays a vital role in embryonic development, tissue regeneration, and tumor progression. In the kidney, HGF is found in mesenchymal cells, while MET is expressed in non-mesenchymal cells. Activation of the HGF/MET signaling axis is associated with the development of renal cysts (66, 72). Another possible explanation is attributed to the imbalance of testosterone hormone levels, whose secretion decreased under crizotinib treatment but recovered after discontinuation of crizotinib (73). Routine ultrasound and renal function examination are needed when initiating ALK inhibitors.

ALK inhibitors have been associated with the occurrence of acute kidney injury and chronic kidney disease in clinical practice. Acute elevation of serum creatinine when initiating first-generation crizotinib may not be a reflection of true kidney injury, but rather a pseudo-kidney injury due to creatinine transporter inhibition, thereby interfering with creatinine secretion in the proximal tubule (58).

Still, cases of real acute kidney dysfunction are reported in the patients treated with ALK inhibitors. It is thought that MET is mainly expressed in the proximal convoluted tubule, proximal loop of Henle loop and distal convoluted tubule. Crizotinib competitively inhibits creatinine and water secretion by inhibiting the c-Met pathway, which may be the mechanism that predisposes to acute kidney injury (58, 70).

Recently, an analysis revealed that the calculated glomerular filtration rate in cancer patients undergoing TKIs was higher in almost all cases when using cystatin C (74). Therefore, it is important to recalculate with both serum creatinine and cystatin C to identify the actual situation when using crizotinib. Patients undergoing treatment with crizotinib should maintain proper hydration, regularly monitor kidney function, and promptly report any symptoms indicating potential kidney injury to healthcare professionals. Also, physicians should carefully assess the risk-benefit ratio before prescribing crizotinib to patients with known risk factors for kidney disease.

### 3.2 Ceritinib

Ceritinib, an oral small molecule, ATP-competitive tyrosine kinase inhibitor of ALK, is 20 times as potent as crizotinib in enzymatic assays. It is always used in patients harboring ALK mutation involving L1196M, G1296A, I1171T, and S1206Y mutations. The recommended dose of ceritinib is 750 mg orally per day (75, 76). The most common adverse events caused by ceritinib include gastrointestinal disorder (grade 1 or 2 diarrhea, nausea) or hepatic disorders among up to 5% of patients, which generally required essential approach and is reversible after drug modifications or discontinuation. Serum creatinine levels were elevated in 11% of patients, and hypomagnesemia occurred in 8% of patients undergo ceritinib therapy. Patients treated with ceritinib often present with hypophosphatemia and this is dose-dependent (75, 77). Hyponatremia is another common electrolyte disorder in patients treated with ceritinib therapy. More than 5% of patients developed hyponatremia during ceritinib therapy. A postulated explanation attributed hyponatremia in patients treated with ALK inhibitors to inadequate secretion of antidiuretic hormone in the collecting ducts and thus producing hyponatremia (78). Hypocalcemia and hypokalemia are also reported though in patients treated with ceritinib (59).

In conclusion, it is necessary to regularly monitor electrolyte levels and kidney function, recognize symptoms and signs early, and take relevant measures to intervene. Dose reductions have not usually been required (77).

### 3.3 Alectinib

Alectinib, a second-generation oral ALK TKIs, is highly selective and more potent against ALK. It has been used in patients with ALK mutations that cause resistance to crizotinib, such as L1196M and C1156Y (79). The recommended dose of alectinib is 600 mg twice orally per day. Alectinib has good brain barrier penetration, resulting in high concentrations in the cerebral blood fluid. The brain is the most common site of metastasis in patients with ALK-positive NSCLC treated with crizotinib, and many patients suffer recurrence of central nervous system involvement (80, 81). Common adverse effect caused by alectinib are constipation (35.6%), edema (33.6%) and myalgia (30.8%). The gastrointestinal and hepatobiliary disorders are the two most common vital adverse effects that calls for emergent attention and treatment (82).

Still, renal adverse events have been reported in patients treated with alectinib, commonly grade 1 or 2 nephrotoxicity. Grade 3 renal adverse events including elevated serum creatinine and neutropenia were reported in 26% of patients, and hypophosphatemia was reported in 2~4% of patients (81, 83). Ramachandran et al (84) reported that a 72-year-old patient with ALK-positive metastatic NSCLC developed acute kidney injury which necessitated emergency hemodialysis within 6-week treatment of alectinib. He recovered completely within 7-10 days on alectinib withdrawal. Recently, one case of biopsy-proven a mixed pattern of acute interstitial nephritis and acute tubular necrosis was reported with this agent for a 68-year-old patient with ALK-positive NSCLC stage IV. After initiation of corticotherapy, renal function in this patient returned to baseline. The patient was finally discharged from the hospital, the and treatment with alectinib was changed to lorlatinib, and renal function remained stable after 10 months of lorlatinib (85).

### 3.4 Brigatinib

Brigatinib, another highly selective ALK TKIs with a 12-fold stronger potent against ALK than crizotinib, has been widely applied in patients with regressive ALK-positive metastatic NSCLC or resistant to crizotinib. The recommended dose of brigatinib is 90 mg orally per day (86). Gastrointestinal disorder (nausea, diarrhea), fatigue and headache are the most common adverse effects and needed emergent attention and treatment (58).

Brigatinib-induced renal adverse events have been rarely reported in clinical practice. Wang et al (87) reported that a 39-year-old patient with advanced ALK-rearranged NSCLC developed tumor lysis syndrome after 22-day therapy of brigatinib, whose serum creatinine rose from 73 μmol/L to 320.2 μmol/L over 22 days of brigatinib treatment. Emergent hemodialysis is required; however, the patient and his family refused any other further interventions due to the worse prognosis, and finally he passed away within 24h.

### 3.5 Lorlatinib

Lorlatinib, a selective third-generation tyrosine kinase inhibitor, has been used to treat crizotinib-resistant ROS1-positive NSCLC and glioblastoma. Lorlatinib has shown high potency in crizotinib-resistant NSCLC with ROS1 G2032R mutation and the recommended dose of lorlatinib is 100 mg orally per day (88–90). Common adverse events of lorlatinib involve neurocognitive impairments, such as slowed speech and thinking and word-finding difficulty, which are dose-limited (91). Up to 50% of patients develop peripheral edema when treated with lorlatinib (10). Also, lorlatinib-related glomerular-toxicity may happen. Betton et al (92) reported that a 64-year-old female diagnosed with lung adenocarcinoma presented with diffuse edema and proteinuria after 1-month treatment with lorlatinib. She was diagnosed with lorlatinib-related nephrotic syndrome with a kidney-biopsy revealing diffuse podocytes foot process disappearance under electron microscopy and her symptoms relived after discontinuation of this drug. However, due to disease progression, the patient was restarted with lorlatinib and subsequently increased to 3.6 g/g proteinuria within 3 days of initiation (92). In addition, McGee et al (93) described a case of 63-year-old female with lung adenocarcinoma developed extinct hyperlipidemia, which may be caused by minimal change disease under renal biopsy. Another case reported by Lee et al (94) revealed a 68-year-old with ROS1 rearranged stage IV lung adenocarcinoma presented proteinuria after receiving lorlatinib, and her symptoms improved when reducing dose from 100 mg to 50 mg daily. However, the related mechanism remained unknown.

Peripheral edema seems to be the most common adverse event after ALK inhibitor therapy, occurring in nearly 50% of participants under the first-generation crizotinib and the third-generation lorlatinib, generally grade 1-2 adverse events (10). However, the exact mechanism remained unknown, but a postulated mechanism referred to the inhibition of the c-MET pathway. In the kidney, MET is expressed in the tubules, including proximal tubules, proximal loop of Henle and the distal tubules. Inhibition of this pathway may lead to electrolyte disorders (73). Peripheral edema generally appears late in the therapy of ALK inhibitors, which appears to be a late-onset cumulative effect. In patients with low-grade edema, compression stockings, leg elevation, and lifestyle changes should be advised first before starting dose adjustment (58). Also, it is important to monitor renal function regularly and take management with dose modification. Diuretics drugs may be a proper choice for cases with more resistant.

## 4 BRAF inhibitors

BRAF, one of the oncogenic drivers, accelerates the RAS-RAF-MEK-ERK pathway and induces cell growth and proliferation, which plays a critical role in cancer progression (95). Approximately 2-4% of patients with NSCLC have been shown to have mutations in the BRAF gene (96).

Vemurafenib, an oral selective BRAF inhibitor, has been used in treatment against NSCLC and melanoma harboring BRAF mutation (97, 98). Vemurafenib, which is highly protein-bound, is metabolized mainly in the liver and finally excreted in the feces (94%), rarely through urine (1%), so it may be possible to use it in patients with renal insufficiency at its usual doses. Similar to vemurafenib, dabrafenib is highly bound to protein and is widely distributed. However, dabrafenib is a higher drug for renal elimination than vemurafenib (71% fecal excretion and 23% urinary excretion). It can also be used for mild to moderate renal impairment, but the terminology for patients with severe renal impairment is lacking (99).

Proteinuria and decreased glomerular filtration rate are common renal adverse events reported by vemurafenib (100). Launay-Vacher et al (101) reported a case series about 8 patients that glomerular filtration rate decreased varied from 20 to 74% after vemurafenib therapy and concomitant use of other nephrotoxic drug, such as cisplatin and zoledronic acid, and a patient received renal biopsy revealing acute tubular necrosis. Electrolyte disorders are common renal adverse effects in clinical practice. In a phase 2 trial combined dabrafenib and trametinib in patients with BRAFV600E-mutant NSCLC, hypophosphatemia, hyponatremia and hypercalcemia has been reported in patients (102).

The mechanism of renal injury caused by BRAF inhibitors is not well understood, but may be related to their blocking of signal transduction of downstream cellular pathways and increased susceptibility of renal tubules to ischemic injury (103). When treating with dabrafenib and vemurafenib, serum creatinine and electrolytes must be routinely monitored and glomerular filtration rate calculated before the first dose. Still, the mechanism has remained unclear yet. Meanwhile, the associated cases are limited. Dabrafenib-related renal toxicity is limited, but more data and time are needed before concluding that these drugs are not nephrotoxic. Oncologists and nephrologists need to be aware of the nephrotoxicities of these agents.

## 5 VEGF inhibitors

After Folkman first proposed the angiogenesis theory of tumor growth and metastasis, researchers have continued to make intense efforts to target this pathway to stop tumor growth. One of the most effective promoters is involved in the VEGF pathway (104). The most widely used small-molecule drugs included bevacizumab, sorafenib, sunitinib, axitinib, and pazopanib (13). In the kidneys, VEGF is expressed in podocytes and maintains the normal function of the glomerular endothelium (105).

Common renal adverse effects related to VEGF inhibitors in lung cancer therapy include proteinuria, acute renal failure and hypertension (106). Zhu et al (107) have shown that the incidence of all-grade hypertension was 21% and a significantly higher incidence in patients with renal cell carcinoma in a meta-analysis of total 4609 patients treated with sunitinib. In the treatment of lung cancer, bevacizumab is more likely to induce nephrotoxicity than other VEGF inhibitors (108, 109).

Bevacizumab, a recombinant human monoclonal antibody that binds to all known vascular endothelial growth factor A (VEGF-A) subtypes and exerts antiangiogenic effects by blocking the binding of VEGF-A to VEGF receptors (primarily VEGFR-1 and VEGFR-2) on the surface of endothelial cells, as VEGF-A binds to VEGFR-1 and VEGFR-2 to promote endothelial cell proliferation, activate survival pathways, and form new blood vessels. Therefore, bevacizumab plays an important role in the treatment of advanced cancer, including NSCLC (109, 110). The most common renal impairment of bevacizumab is proteinuria in clinical practice (111). In a pooled analysis of bevacizumab-treated patients including patients diagnosed NSCLC, the incidence of proteinuria of any grade was 8.2% and 4.6% in the bevacizumab and control groups, respectively, while the incidence of grade 3/4 proteinuria was 1.4% and 0.2%, respectively (108). Another population-based retrospective cohort study revealed that patients treated with bevacizumab had a significantly 1.35-fold higher risk of CKD than those who did not receive bevacizumab (109).

The main mechanisms related to proteinuria may include the interference with podocytes endothelial VEGF axis signaling, increased intraglomerular pressure caused by secondary hypertension and subacute glomerular thrombotic microangiopathy. The most common histopathological manifestations of the kidney caused by VEGF inhibitors is thrombotic microangiopathy, followed by glomerular lesions and interstitial nephritis (112, 113).

Inhibition of the VEGF pathway may lead to hypertension. A possible mechanism is that VEGF is a mediator of endothelium-dependent vasodilation, leading to upregulation of nitric oxide synthase. Meanwhile, capillary rarefaction and increased prostacyclin production play a role in exacerbating hypertension (112, 114). Monitoring of proteinuria during treatment is also important, especially during the treatment with bevacizumab. Patients with proteinuria ≥2.0 g/24 h need to suspend the medication. And if there is a proteinuria > 3.0 g/24 h or if nephrotic syndrome occurs, permanent discontinuation of the medication is required. When this happens, we may consider switching to other drugs. For example, in patients with nephrotic syndrome associated with gefitinib therapy, we may consider erlotinib as a potential treatment option. Acute kidney injury and other renal dysfunction may occur during the treatment, so creatinine and urea levels should be determined. Increased monitoring frequency is required when creatinine levels increase >1 to 1.5-fold baseline. If the creatinine concentration increases 1.5-fold baseline, administration of methylprednisolone is required. A renal biopsy should also be considered. The administration of drugs should be delayed when creatinine levels increase >3-fold baseline (111).

## 6 Discussion

In targeted therapy for lung cancer, especially EGFR and ALK inhibitors, may damage the glomeruli, renal tubules or any other part of the nephron, leading to deterioration of renal function and the appearance of various clinical symptoms such as AKI, electrolyte disturbances, nephrotic syndrome and glomerulonephritis (12). EGFR was expressed in the kidney, so EGFR inhibitors may cause AKI, renal failure or renal impairment. Mutation of EGF gene also resulted in the activation of TRPM-6 type channel and thus hypomagnesemia occurred. In addition, EGFR inhibitors may cause hypophosphatemia and hypocalcemia by affecting the Na/Pi (sodium-phosphate) co-transporter channels (26). The ALK inhibitors may cause peripheral edema by inhibiting the c-MET pathway and associate with an acute increase in serum creatinine by inhibiting of a creatinine transporter, thus interfering with the secretion of creatinine in the proximal tubule (73).

The kidney is vulnerable to injury from the targeted therapy used to treat lung cancer, so we recommend routine physical examinations, imaging, serum or urine monitoring in these patients. It is important to improve awareness of the factors that that enhance nephrotoxic risk. These factors include specific patient characteristics, nephrotoxicity of the substance itself and renal handling of the causative substance. Electrolyte disorders including hypophosphatemia, hyponatremia, hypokalemia and hypocalcemia can be life-threatening.

Therefore, it is advisable to check electrolyte levels prior to the initiation of therapy, and then routinely monitor the electrolyte levels to prevent the development of severe complications caused by electrolyte disorders. Drugs should not be discontinued unless the symptoms are very severe.

## 7 Conclusion

In recent years, significant progress has been made in the early diagnosis and treatment of cancer, which has a significant impact on prolonging the survival of patients. However, nephrotoxicity may happen after initiation of the therapy and this may force reduction or discontinuation of the medication. Nephrotoxicities induced by targeted therapy cause manifestations of various forms, ranging from nephrotic syndrome to acute kidney injury. Currently, there is limited data on many kidney-related toxicities, especially new drugs released in recent years. Yet, the relevant mechanism has remained unclear. It is necessary to further study the potential mechanism of the effect of these drugs on the kidney. It is important to monitor the renal function when initiating the targeted therapy and pay close attention and regular follow-up. In the future, further studies should be carried out and animal experiments are needed to explore the potential mechanism of these drugs on the kidney and reduce renal adverse events.

## Author contributions

QL: Conceptualization, Writing – original draft, Writing – review & editing, Methodology, Resources, Validation. JL: Conceptualization, Methodology, Resources, Validation, Writing – original draft, Writing – review & editing. GH: Conceptualization, Writing – review & editing. AX: Conceptualization, Writing – review & editing. SL: Conceptualization, Writing – review & editing. BT: Conceptualization, Writing – review & editing.
